# Autoimmune hypophysitis presenting with intracranial multi-organ involvement: three case reports and review of the literature

**DOI:** 10.1186/1756-0500-6-560

**Published:** 2013-12-28

**Authors:** Atsushi Kanoke, Yoshikazu Ogawa, Mika Watanabe, Toshihiro Kumabe, Teiji Tominaga

**Affiliations:** 1 Department of Neurosurgery, Kohnan Hospital, 4-20-1 Nagamachiminami, Taihaku-ku, Sendai 982-8523, Miyagi, Japan; 2 Department of Pathology, Tohoku University Graduate School of Medicine, Sendai, Miyagi, Japan; 3 Department of Neurosurgery, Tohoku University Graduate School of Medicine, Sendai, Miyagi, Japan

**Keywords:** Hypophysitis, Hypothalamus, Immunoglobulin G4-related disease, Severe internal carotid artery stenosis, Steroid pulse therapy

## Abstract

**Background:**

Autoimmune hypophysitis very rarely spreads to nearby organs outside the pituitary tissue, for unknown reasons, with only 5 reported cases of hypophysitis spreading over the cavernous sinus.

**Case presentation:**

Three patients presented with cases of non-infectious hypophysitis spreading outside the pituitary tissue over the cavernous sinus. All three cases were diagnosed with histological confirmation by transsphenoidal surgery, and the patients showed remarkable improvement with postoperative pulse dose steroid therapy, including disappearance of abnormal signal intensities in the bilateral hypothalami on magnetic resonance imaging, resolution of severe stenosis of the internal carotid artery, and normalization of swollen pituitary tissues. Two of 3 cases fulfilled the histological criteria of immunoglobulin G4-related disease, although none of the patients had high serum immunoglobulin G4 level.

**Conclusion:**

The true implications of such unusual spreading of hypophysitis to nearby organs are not fully understood, but the mechanism of occurrence might vary according to the timing of inflammation in this unusual mode of spreading. Pulse dose steroid therapy achieved remarkably good outcomes even in the patient with progressive severe stenosis of the internal carotid artery and rapid visual deterioration.

## Background

Autoimmune hypophysitis is classified into subtypes based on the histological findings into granulomatous hypophysitis, lymphocytic hypophysitis, and xanthomatous hypophysitis, or based on the site of involvement or endocrinological behavior into adenohypophysitis, infundibulo-neurohypophysitis, and panhypophysitis [1]. Definitive autoantibodies are not often detected, and these subtypes may overlap [2]. The former classification is based on the predominance of inflammatory cells, and so could be the same inflammatory disease in different stages of healing [3,4]. Therefore, the specific characteristics and relationships between the subtypes of autoimmune hypophysitis should be clarified and re-established. The intracranial inflammatory process very rarely spreads outside the pituitary tissue, except for a few cases of presumed immunoglobulin G4 (IgG4)-related pachymeningitis [5-8]. Only 5 cases of hypophysitis spreading over the cavernous sinus have been reported [9-13].

In this report we describe three cases of autoimmune hypophysitis spreading to nearby organs from outside the pituitary tissue over the cavernous sinus, and discuss the limitation and problems underlying the classification of hypophysitis from the histological and endocrinological aspects.

## Case presentation

### Case 1

**Figure 1:**
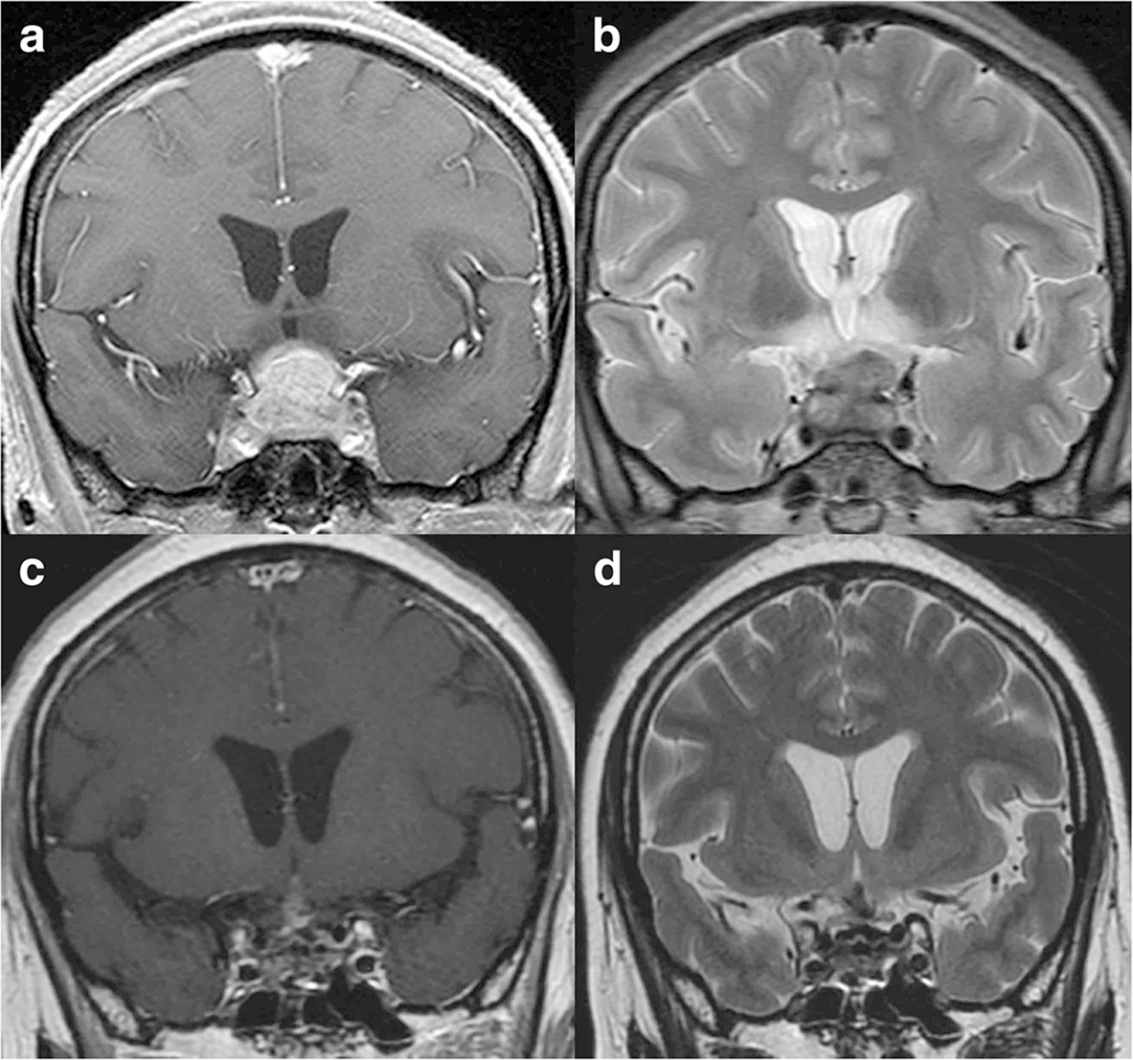
**Chronological MR images of Case 1. (a)** Preoperative coronal T1-weighted MR image with gadolinium revealing a sellar lesion extending to the suprasellar cistern and bilateral cavernous sinuses. **(b)** Preoperative coronal T2-weighted MR image revealing a sellar lesion extending to the suprasellar cistern and a vast irregular high intensity area in the bilateral hypothalami. **(c, d)** Coronal T1-weighted MR image with gadolinium **(c)** and T2-weighted MR image **(d)** showing significant reduced volume of the lesion and disappearance of abnormal intensity in the bilateral hypothalami at 13 months after operation.

A 60-year-old woman was referred to our hospital because of drastically decreased visual acuity of the left eye. She had been treated for previous hypothyroidism with 100 μg levothyroxine daily. On admission, neurological examination detected three-fourths visual field defect of the left eye with severely decreased visual acuity. Endocrinological examinations showed low levels of serum cortisol and thyroid-stimulating hormone (TSH), and hyperprolactinemia (Table 1). Magnetic resonance (MR) imaging revealed a large sellar lesion, which extended to the suprasellar cistern, and was heterogeneously enhanced after gadolinium administration (Figure 1a). The optic chiasm was considerably displaced upward, and T2-weighted MR imaging showed vast irregular high intensity areas in the bilateral hypothalami (Figure 1b).

**Table 1:** Serial endocrine values of the 3 patients

**Case no.**	**ACTH**	**Cortisol**	**TSH**	**FT3**	**FT4**	**LH**	**FSH**	**GH**	**PRL**	**IgG4**
	**(pg/ml)**	**(μg/ml)**	**(μIU/ml)**	**(pg/ml)**	**(ng/ml)**	**(mIU/ml)**	**(mIU/ml)**	**(ng/ml)**	**(ng/ml)**	**(mg/dl)**
1	10.0→	<1.0↓	<0.01↓	3.45→	1.37→	<0.10↓	1.53→	2.96↑	15.93↑	8.0→
2	45.5→	9.3→	0.627↓	1.22↓	0.68↓	0.39↓	3.30→	0.59→	42.2↑	13.5→
3	-	<0.64↓	0.47→	1.41↓	0.60↓	0.80↓	0.40↓	-	4.80→	-

*ACTH*, Adrenocorticotropic hormone; *TSH*, Thyroid-stimulating hormone; *FT3*, Free tri-iodothyronine; *FT4*, Free thyroxine; *LH*, Luteinizing hormone; *FSH*, Follicle-stimulating hormone; *GH*, Growth hormone; *PRL*, Prolactin; *IgG4*, Immunoglobulin G4.

→ indicates within the normal ranges; ↓ indicates lower than normal limits; ↑ indicates higher than normal limits.

Bar means unevaluated.

Transsphenoidal surgery was performed. The dura mater was thickened to 2 mm, and yellowish gum-like tissue had filled the sella. Intraoperative rapid diagnosis suggested inflammatory disorder with no indications of adenoma-like change or malignant tumor, so surgery was limited to biopsy. Pulse dose steroid therapy was started in the immediate postoperative period using 1000 mg methylprednisolone initially for 3 days, then tapered and changed to oral intake of 0.5 mg dexamethasone daily. MR imaging at 10 days after the treatment showed the lesion was reduced in volume to 60% and the abnormal high intensity areas in the bilateral hypothalami had disappeared. Her visual field defect had improved to partial bitemporal hemianopsia. She was discharged with daily administration of 0.5 mg dexamethasone. MR imaging at 13 months after the treatment showed the lesion had decreased in size resulting in only a slightly swollen pituitary stalk (Figure 1c, d).

**Figure 2:**
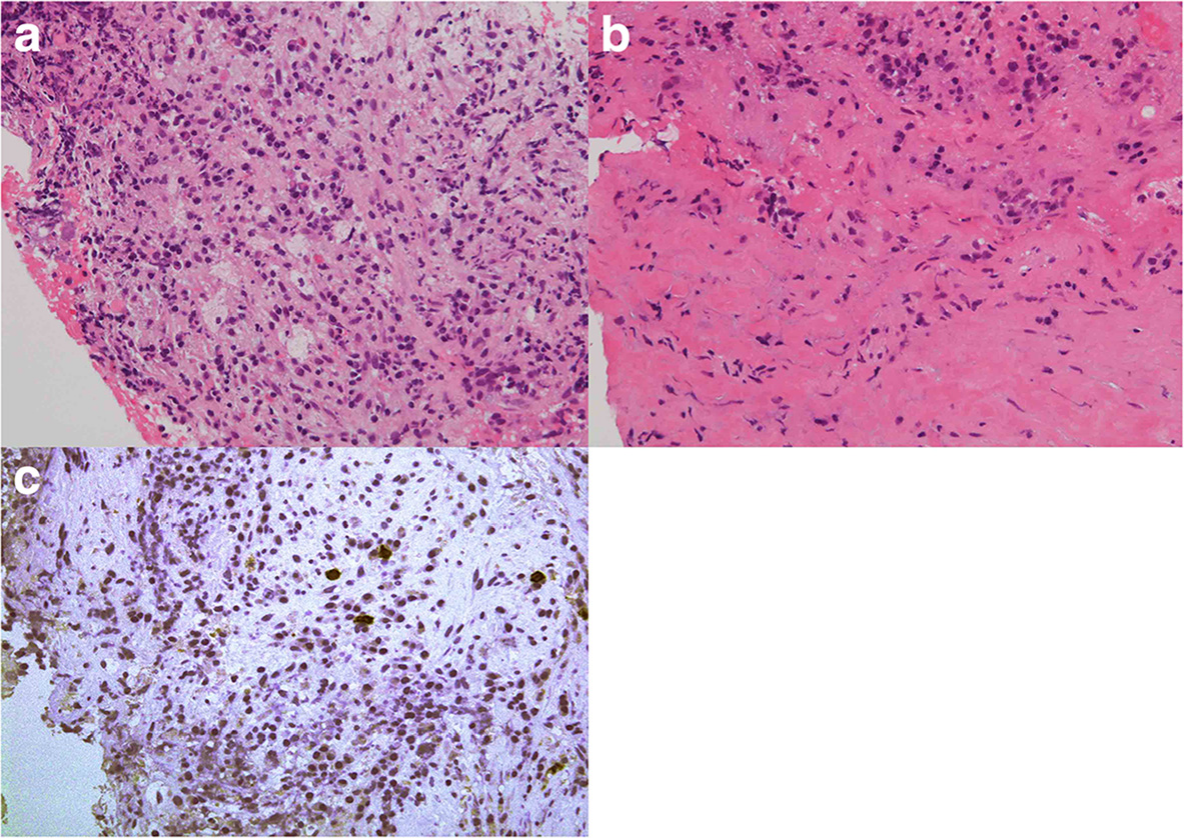
**Photomicrographs of Case 1. (a, b)** High-power photomicrographs showing inflammatory cells infiltrating the pituitary lesion **(a)** and the dura mater **(b)**. Hematoxylin and eosin staining, original magnification × 200. **(c)** Immunohistochemical staining showing infiltrated plasmacytes positive for IgG4 in the pituitary tissue. Original magnification × 200.

Histological examination of the biopsy specimen showed massive infiltration of plasma cells and lymphocytes, and increased collagenous fiber and mesenchymal spindle cells. Staining for IgG4 was positive in the cytoplasm of approximately 10% of invaded plasma cells. Massive infiltration of similar inflammatory cells was detected in the dura mater (Figure 2). The histological diagnosis was autoimmune hypophysitis. The serum IgG4 concentration was within the normal range. This case was thought to represent hypophysitis spreading to the dura mater and hypothalamus.

### Case 2

**Figure 3:**
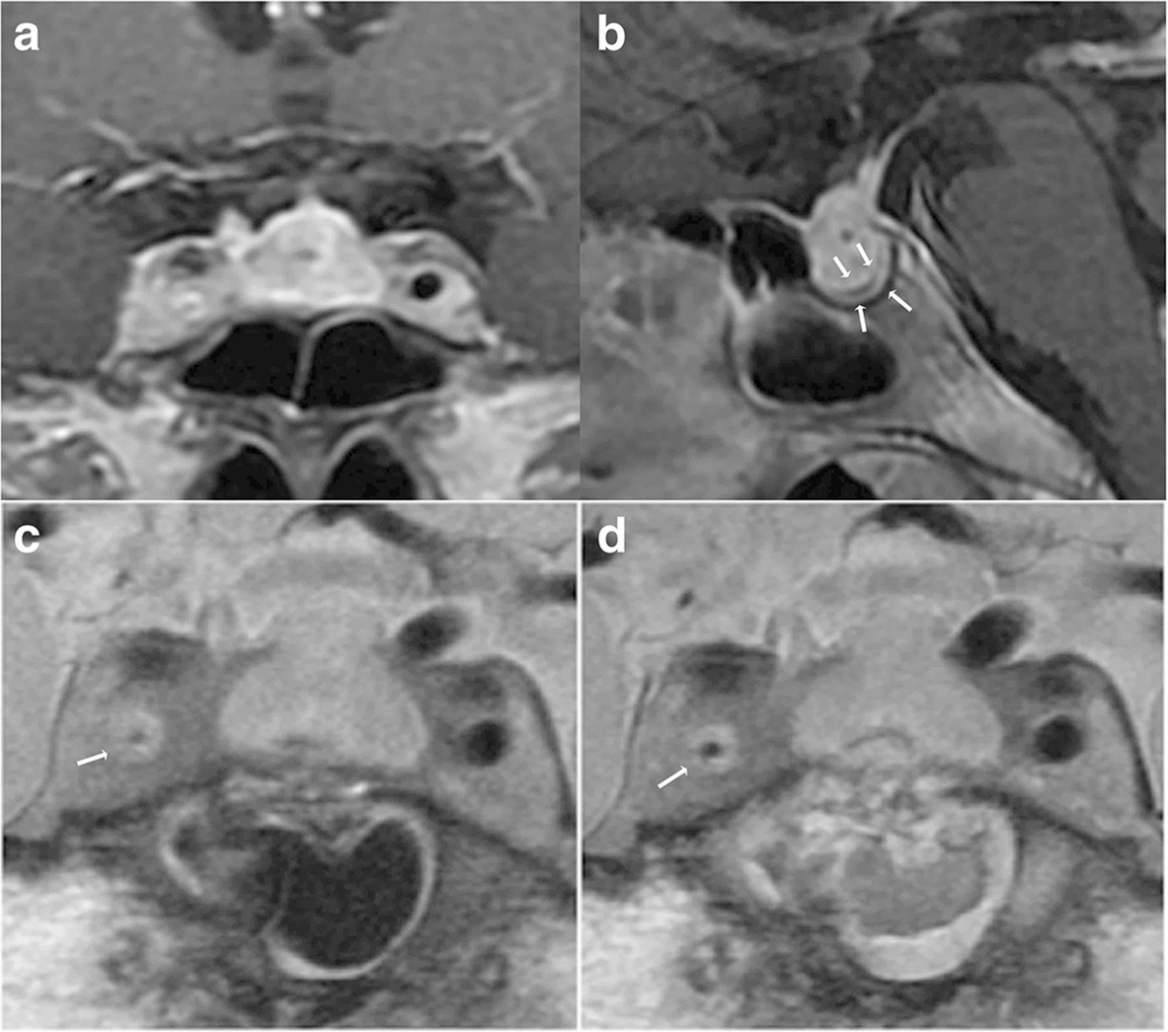
**Chronological MR images of Case 2. (a, b)** Preoperative sagittal T1-weighted MR images with gadolinium revealing a sellar lesion extending to the suprasellar cistern **(a)**, and markedly thickened dura mater **(b)**. **(c)** Preoperative coronal plaque MR image revealing the thickened wall of the right internal carotid artery and the severely narrowed lumen. **(d)** Postoperative coronal plaque MR image showing improved right internal carotid artery stenosis.

A 53-year-old woman was referred to our hospital because of severe dull headache and heavy feeling in the retroorbital space. No other neurological findings were detected and endocrinological studies showed disturbed TSH-thyroid axis with hyperprolactinemia (Table 1). MR imaging revealed a significantly enhanced en bloc mass lesion extending over the pituitary gland, cavernous sinus, and basal dura mater (Figure 3a, b). The wall of the right internal carotid artery was thickened and the lumen was severely narrowed with diameter of 0.8 mm (Figure 3c).

Transsphenoidal surgery was performed. The dura mater was thickened to 3 mm, and the pituitary gland was edematous. Intraoperative rapid diagnosis suggested inflammatory disorder with massive infiltration of lymphocytes, so surgery was limited to biopsy. Pulse dose hydrocortisone treatment was started from the day of the surgery, using intravenous 200 mg hydrocortisone initially and tapered gradually. MR imaging 3 days after the operation showed regression of the right internal carotid artery stenosis to 1.4 mm (Figure 3d), and severe dull headache and heavy feeling in the retroorbital space had disappeared. She remained in good condition for 6 months while taking 0.5 mg dexamethasone daily.

**Figure 4:**
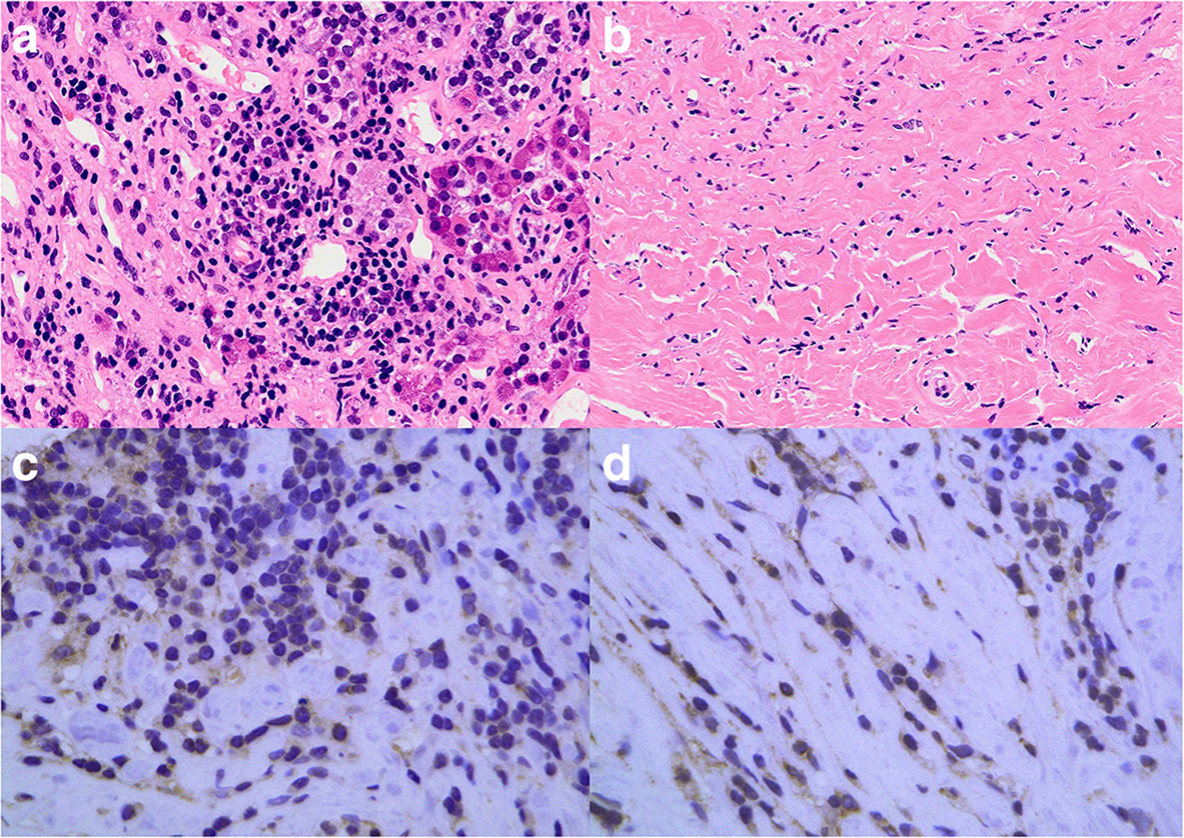
**Photomicrographs of Case 2. (a, b)** High-power photomicrographs showing inflammatory cells infiltrating the pituitary tissue **(a)** and the dura mater **(b)**. Hematoxylin and eosin staining, original magnification × 200. **(c, d)** Immunohistochemical staining showing infiltrated plasmacytes positive for IgG4 in the pituitary tissue **(c)** and the dura mater **(d)**. Original magnification × 400.

Histological examination of the biopsy specimen showed massive infiltration of plasma cells and the dura mater was fibrously thickened with massive infiltration of similar inflammatory cells. Staining for IgG4 was positive in the cytoplasm of approximately 50% of invaded plasma cells (Figure 4). The histological diagnosis was autoimmune hypophysitis. Serum IgG4 concentration was within the normal range. This case was thought to represent hypophysitis spreading to the dura mater, cavernous sinus, and wall of the internal carotid artery.

### Case 3

**Figure 5:**
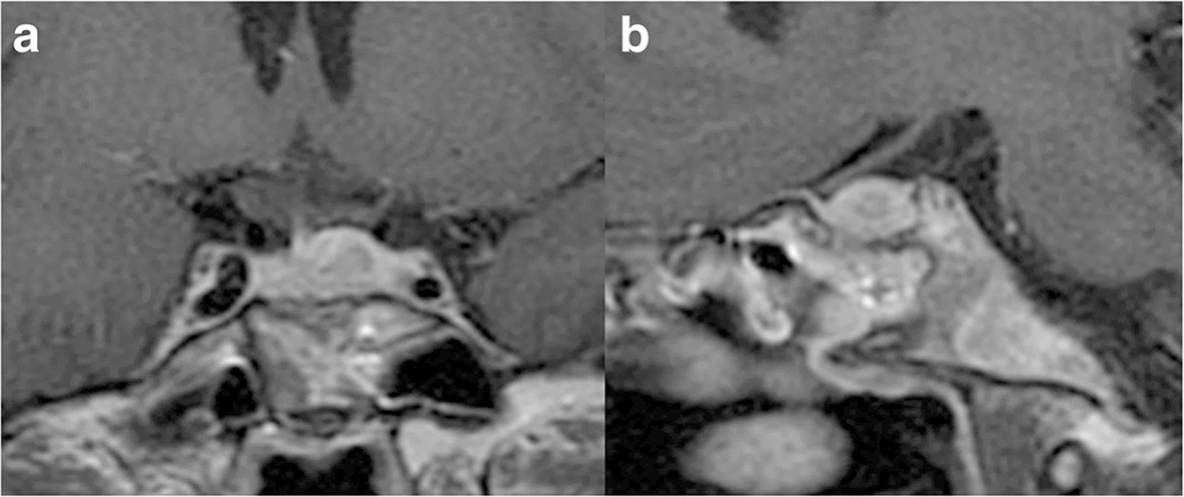
**Preoperative MR images of Case 3.** Preoperative coronal **(a)** and sagittal **(b)** T1-weighted MR images with gadolinium revealing a sellar lesion enhanced heterogeneously with less enhancement in the central portion of the lesion.

A 27-year-old woman had suffered from amenorrhea for 12 years and presented with deteriorated visual function and mild diabetes insipidus. She underwent transsphenoidal biopsy for suprasellar tumor. Histological examination with hematoxylin-eosin and periodic acid-Schiff staining disclosed germinoma with typical two-cells pattern (not shown). She received systemic chemotherapy with bleomycin, etoposide, and cis-diamminedichloroplatinum, and irradiation to the tumor bed (20 Gy). After completion of the initial treatment, MR imaging revealed complete resolution of the tumor. She had remained in good condition with hormonal replacement therapy, including dexamethasone, levothyroxine, and desmopressin acetate daily. No abnormal findings were seen at follow-up MR imaging examinations, which were performed every 6 months thereafter. Six years after the first therapy, she suffered headache and easy fatigability. Endocrinological examinations showed panhypopituitarism and hypothyroidism (Table 1). MR imaging showed a sellar lesion extending to the suprasellar cistern, and recurrence of germinoma was suspected (Figure 5).

**Figure 6:**
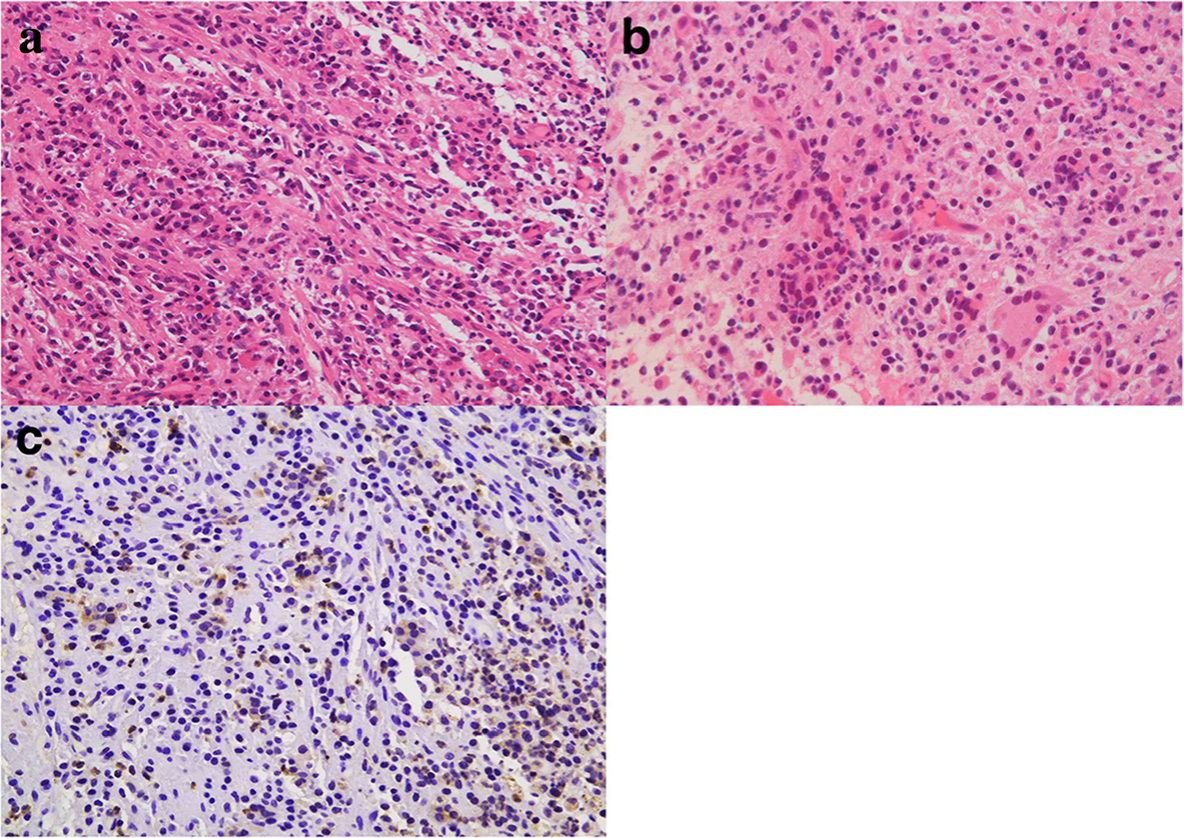
**Photomicrographs of Case 3. (a, b)** High-power photomicrographs showing inflammatory cells infiltrating the pituitary tissue **(a)**, which appeared to be chronic inflammatory lesions with prominent histiocytes and corresponded to nonspecific granulomatous inflammation, not epithelioid granuloma as seen in sarcoidosis, and the extradural tissue **(b)**. Hematoxylin and eosin staining, original magnification × 200. **(c)** Immunohistochemical staining showing infiltrated plasmacytes positive for IgG4 in the pituitary tissue. Original magnification × 200.

Second surgery was again performed through the transsphenoidal approach. Intraoperative rapid diagnosis suggested inflammatory disorder with massive infiltration of lymphocytes in the pituitary tissue, dura mater, and extradural sphenoidal mucosa, so surgery was limited to biopsy. Pulse dose hydrocortisone treatment was started from the day of the surgery using intravenous 200 mg hydrocortisone initially and tapered gradually. Histological examination of the biopsy specimen showed massive infiltration of inflammatory cells including lymphocytes in both the internal and external tissue of the dura mater. Staining for IgG4 was positive in the cytoplasm of approximately 40% of invaded plasma cells (Figure 6). The histological diagnosis was granulomatous hypophysitis. This case was thought to represent hypophysitis spreading to the extradural sphenoidal tissue.

## Discussion

Lymphocytic hypophysitis spreading to nearby organs is rarely reported. However, most cases are located in the cavernous sinus, and are only presumed diagnoses based on the clinical symptoms such as diplopia [14-17]. Spread to other nearby organs has been described in only 5 cases reported since 1985, to the wall of internal carotid artery in 3 cases, the hypothalamus in one, and the sphenoidal mucosa in one [9-13] (Table 2). Nine cases of hypertrophic pachymeningitis with intraaxial inflammatory involvement have been reported [5,6,18], in which inflammatory cells were presumed to have infiltrated the brain parenchyma after invading the subarachnoid and Virchow-Robin spaces, resulting in pachymeningoencephalitis. The same inflammatory mechanism may have been involved in Case 1.

**Table 2:** Reports of autoimmune hypophysitis with extra-pituitary spread over the cavernous sinus

**Literature**	**Age/sex**	**Chief complaint**	**Organ of inflammatory spread**	**Remarks**
Fujisawa, 2009 [9]	49/F	Diabetes insipidus, retroorbital pain, diplopia, anterior pituitary hypofunction	Bilateral ICA occlusion	Steroid therapy
Demetri et al., 2010 [10]	29/F	Headache, visual disturbance	Isolated sphenoidal mucosa	Subtotal removal
Melgar et al., 2006 [11]	38/F	Severe headache, cerebral ischemia	Bilateral ICA occlusion	ECA-ICA bypass
Thodou et al., 1995 [12]	18/F	Severe headache, diabetes insipidus	Basal hypothalamus	
Zoeller et al., 2012 [13]	41/M	Optic neuritis, fatigue, loss of libido	Unilateral ICA stenosis	Past history of optic neuritis, steroid
Present Case 1	60/F	Rapid visual deterioration, masked diabetes insipidus	Bilateral hypothalami	Steroid pulse therapy
Present Case 2	53/F	Severe retroorbital pain	Unilateral ICA stenosis	Steroid pulse therapy
Present Case 3	27/F	Headache, fatigue, diabetes insipidus	Extradural sphenoidal mucosa	Past history of germinoma, steroid pulse therapy

*F*, Female; *M*, Male; *ICA*, Internal carotid artery; *ECA*, External carotid artery.

Very few reports have described hypophysitis spreading to the surrounding structures over the cavernous sinus. Consequently, the current classification cannot accommodate any of our 3 patients into the accepted categories of adenohypophysitis, infundibulo-neurohypophysitis, or panhypophysitis. In contrast, non-bacterial inflammatory spread to multiple organs sometimes occurs from extracranial lesions, and IgG4-related pancreatitis frequently spreads to extrapancreatic organs such as the bile ducts, collecting renal duct, or retroperitoneal space [19]. The underlying mechanisms of such unusual spread of hypophysitis remain unclear, but the relationship between peripheral blood and local tissue may vary according to the timing of inflammation. A new category of hypophysitis has been proposed which involves an IgG4-related mechanism [20]. Like our cases, the spread of hypophysitis may have some relation to the high concentrations of IgG4-positive plasma cells in the local tissue. We hope that further experience will clarify the specific characteristics and the relationships of our cases with the current classification of hypophysitis.

Transsphenoidal surgery has been performed as the treatment for hypophysitis in most cases, and total removal of the inflammatory process continues to be recommended for patients with visual deterioration. Steroid therapy was found to be less effective in some cases [21], but a significant effect could be obtained within only 1 week even in patients with critical visual status such as our Case 1 with administration of massive doses of methylprednisolone [22]. Therefore, if hypophysitis is suspected based on neuroimaging findings or clinical symptoms, transsphenoidal surgery is recommended for histological confirmation. However, great care is needed to establish the diagnosis using a small biopsy specimen [23]. If intraoperative rapid diagnosis suggests inflammatory disorder, pulse dose steroid therapy should be initiated. Re-operation for total removal should be performed if deterioration occurs, as obtaining an adequate specimen is important because hypophysitis with infiltration of inflammatory cells can be confused with granulomatous reaction of neurohypophyseal germinoma [24].

## Conclusion

The present three cases of autoimmune hypophysitis spread outside the pituitary tissue with severe fibrosis. Pulse dose steroid therapy achieved remarkably good outcomes even in the patient with progressive severe stenosis of the internal carotid artery and rapid visual deterioration.

### Ethics

The therapeutic protocol was approved by the internal ethics committee of Kohnan Hospital 2012.

## Consent

Written informed consent was obtained from the three patients for publication of this case report and accompanying images. A copy of the written consent is available for review by the Editor-in-Chief of this journal.

## Abbreviations

IgG4: Immunoglobulin G4; MR: Magnetic resonance; TSH: Thyroid-stimulating hormone.

## Competing interests

The authors declare that they have no competing interests.

## Authors’ contributions

AK analyzed the patient data regarding the endocrinological outcome, and was a major contributor in writing the manuscript. YO performed transsphenoidal biopsy of all the patients. MW performed pathological examinations. TK performed tumor removal in the first surgery of Case 3. TT gave an essential suggestion and supervised this manuscript. All authors read and approved the final manuscript.
